# High Biofidelity 3D Biomodel Reconstruction from Soft and Hard Tissues (Knee), FEM, and 3D Printing: A Three-Dimensional Methodological Proposal

**DOI:** 10.1155/2021/6688164

**Published:** 2021-04-03

**Authors:** Rodrigo Arturo Marquet-Rivera, Guillermo Urriolagoitia-Sosa, Rosa Alicia Hernández-Vázquez, Beatriz Romero-Ángeles, Octavio Alejandro Mastache-Miranda, Guillermo Urriolagoitia-Calderón

**Affiliations:** ^1^Instituto Politécnico Nacional, Escuela Superior de Ingeniería Mecánica y Eléctrica, Sección de Estudios de Posgrado e Investigación, Unidad Profesional Adolfo López Mateos “Zacatenco”, Avenida Instituto Politécnico Nacional s/n, Edificio 5, 2do. Piso, Col, Lindavista, C.P. 07320 Ciudad de, Mexico; ^2^Universidad Politécnica del Valle de México, Departamento de Mecatrónica, Av. Mexiquense s/n esquina Av. Universidad Politécnica, Col. Villa Esmeralda, Tultitlán, C.P. 54910 Estado de, Mexico

## Abstract

The modelling of biological structures has allowed great advances in Engineering, Biology, and Medicine. In turn, these advances are seen from the design of footwear and sports accessories, to the design of prostheses, accessories and rehabilitation treatments. The reproduction of the various tissues has gone through an important evolution thanks to the development of computer systems and programs. However, knowledge of the medical-biological and engineering areas continues to be required, and it involves a considerable investment of time and resources. The resulting biomodels still require great precision. The present work shows a methodology that allows to optimize computational resources and reduce elaboration time of biomodels. Through this methodology, it is possible to generate a biomodel of high biofidelity of a human knee. This biomodel is constituted by hard tissues (cortical and trabecular bones) and soft tissues (ligaments and meniscus) resulting in the modelling of the lower third of the femur, the tibial plateaus, the anterior cruciate ligament, posterior cruciate ligament, external lateral ligament, interior lateral ligaments, and the meniscus. With this model and methodology, it is possible to perform numerical analyses that will provide results very similar to those of real life. As, the methodology allows to assign the mechanical properties to each tissue and the anatomical structure.

## 1. Introduction

There are many computer programs that allow their application to any scientific and/or technical discipline of everyday life. In the specific case of Biomechanics, there are computational programs commonly used for Engineering, Biology, and Medical areas, to produce three-dimensional models of the structural components of the human body, such as soft and hard tissues (muscles, tendons, bones, ligaments, veins, etc.). In these three-dimensional models, the Engineering programs are applied for the assignment of the mechanical attributes of the tissues, as they are applied in any material. In addition, through them, it is possible to perform numerical simulations using the Finite Element Method (FEM) [1]. Nowadays, it is possible to emerge with new knowledge on the behavior of the human body when facing diverse external agents and situations of the daily life [2, 3].

The models are developed from studies of imaging, a branch of medicine responsible for obtaining images of the internal components of the body. Imaging covers a wide range of techniques and studies [4]. However, for the generation of biomodels, two specific types of studies are used: computational tomography (CT) and magnetic resonance imaging (MRI) [5]. In both studies, cuts of a longitudinal section of the member to be studied are obtained; with a minimum distance between them, from these cuts a series of two-dimensional images are obtained. Then, when are placed together, form the three-dimensional element [6]. It is clear that in performing this operation, it is necessary a coarse knowledge of both, the computational tool to be used and the anatomy of the studied object. This is because, although the images obtained with the techniques are extremely precise, there are a large number of structural elements, typical of the human body that can be confused or not clearly appreciated on their limits. So, if a bad calibration of the imaging equipment occurs, the results obtained would not be precise.

The files obtained after conducting these studies have a DICOM extension (Digital Imagine Communications on Medicine), so it is necessary to use computational tools capable of interpreting this extension [7]. The computer programs that interpret the DICOM extension can generate files with an STL extension that are finally interpreted in computer programs of the CAD/CAM type (Computer Aided Design/Computer Aided Manufacturing), which belong to the area of Engineering. It is possible to import these results into the programs that use the Finite Element Method (FEM). This technique allows predictive and corrective analysis, with the advantage of being non-invasive for the human body. These models are associated with Biomechanics, because they integrate the biological-medical part of the imaging to the mechanical engineering science with the application of an external agent such as forces, movements, restrictions, and materials. For the physiotherapeutic area, this tool can be extremely useful, since it allows the creation and understanding of the required therapy for the rehabilitation of the damaged elements.

In the images obtained by CT or MRI, it is possible to appreciate that each section of the image is subdivided into a small box called a pixel. The pixel is a spatial fraction within a biaxial or 2D dimension. By joining a series of pixels in the different dimensions, it is possible to form a cube or a three-dimensional (3D) object with which a voxel is created. A voxel can also be found in the literature as a volumetric pixel. The voxels can be seen by their grey scale, and by this means, it is possible to determine the density of the element with the help of the Hounsfield units (UH) [8, 9]. Hounsfield units refer to a scale that is used to measure the density of human tissues, with the help of computerized tomography and magnetic resonance imaging equipment. The scale of the Hounsfield units goes from -1000 to 1000 while the gray scale goes from 0 to 255, where the value 0 is a completely obscured pixel and the value 255 is a blank pixel [10]; by means of this coloration (white to black), it is possible to determine the density of the elements of biological tissues without the need to perform physical tests [11, 12].

Imaging is a tool that has provided great support to the medical area, since its application has allowed obtaining a diagnosis with greater precision, helping to perform a better planning of the indicated surgical interventions, among other advantages. On the other hand, in the area of Engineering and Computer Aided Design, it is common the processing of images for the realization of parts, designs, assemblies, or plans before manufacturing through computer programs. The same principle occurs for Biomechanics. When designing a prosthesis, implant, or graft to be embedded in the body, it is necessary to know the whole of the morphology of anatomical entities involved, in order to achieve an optimal coupling between the pieces being designed [13]. On the other hand, anatomical biomodels represent a potential tool for a better diagnosis, treatment, and physiotherapy plans [14, 15].

The objective of this work is to propose a methodology with which better biomodels are obtained to perform numerical analysis of greater precision, better surgical planning, prosthetic designs, 3D printing, and better manipulation of the biomodel.

## 2. Materials and Methods [16]

**Figure 1:**
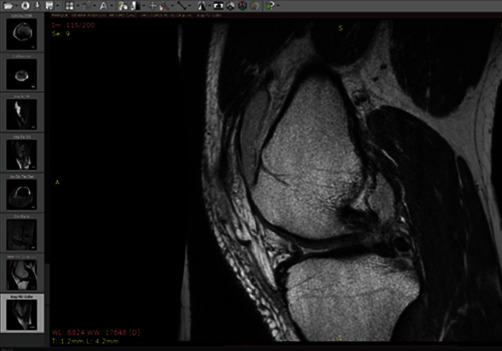
Sequence of cuts of a knee by means of an MRI.

The present work, for the realization of the computational model of the knee, is based on an MRI of the lower limb. From this, a sequence of cuts or images is obtained in a DICOM file (Figure 1), which allows to develop 3D models of the different biological tissues of the human being. The ScanIP® computer program was used, in which the images with DICOM format are imported. This computer program generates a cloud of points over a specific area. After the generation of this cloud of points, it is processed to solidify it, and finally, the three-dimensional structure of the form is obtained; the image of this is shown below.

**Figure 2:**
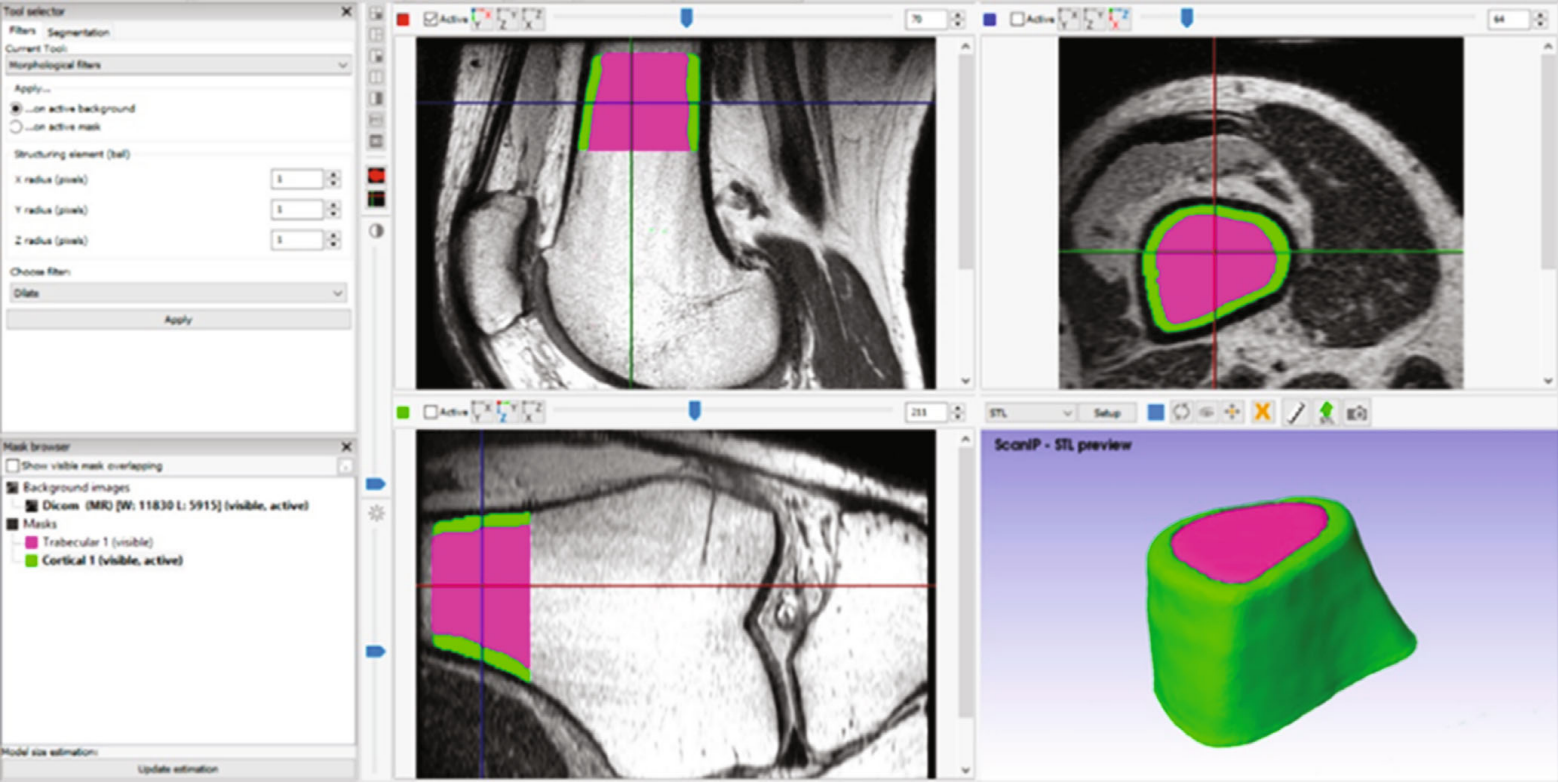
Selection, visualization of the 3 planes and 3D area, filling of each tissue, and three-dimensional visualization.

After carrying out the imaging study and obtaining the MRI, the file is imported into the computer program in DICOM format. Once the image package has been imported into the computer program, the parameters of the tissue to be modelled and the work area are defined. This operation reduces the computational weight of the file, which does not occupy the totality of the computer's memory, as it is advisable to save the computational resource. Once it has been selected the cut to work with, it is delimited, filled, or drawn; in a sequence of superimposed layers, this generates a layer of points that when put one on other in an orderly sequence, it is possible to generate the three-dimensional model. In this way, the different tissues that are needed produce new masks in the file (Figure 2).

This program has a set of tools capable of generating 3D models in .STL (standard triangulation language) format from imaging (magnetic resonance or computational tomography). To perform the segmentation of the images, the threshold tool is used. This computational algorithm uses the density ranges of each pixel (depending on its gray scale or Hounsfield units) and automatically segments the structures of biological tissue. In this way, the adjacent or nontissue structures are discriminated, allowing the biomodel to be made separately for each biological element. Subsequently, the program recognizes the areas defined in each slide and generates a virtual model which is a reference for the solid model. For this, it is necessary to have an upper and lower threshold value, since each segment contains only the pixels with the value within the threshold. A low threshold value corresponds to soft tissue while hard or denser tissues fall into a high threshold, which is possible by means of a histogram which shows Hounsfield units (HU) [17].

**Figure 3:**
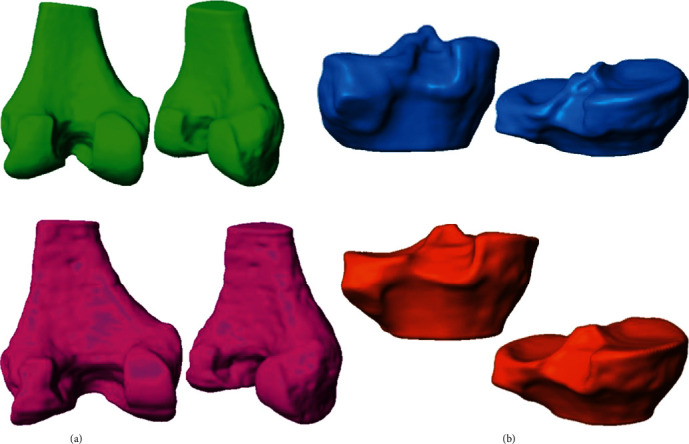
3D biomodels, hard tissues: (a) cortical bones; (b) trabecular bones.

For the knee biomodel generated in this work, the masks corresponding to hard tissues (cortical and trabecular bone) and soft tissues (ligaments and meniscus) were elaborated. The first tissue that is made is the cortical bone of the last third of the knee, that is, the area of the femoral condyles and the first third of the tibia and fibula, in the tibial plateau (Figure 3(a)). Trabecular bone follows, from the aforementioned elements (Figure 3(b)).

**Figure 4:**
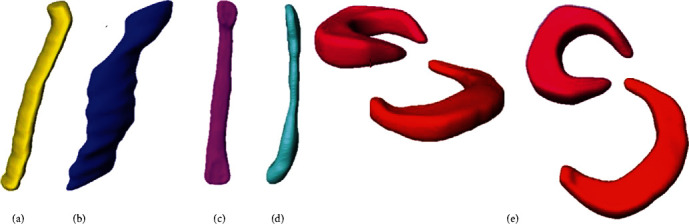
3D biomodels, soft tissues: (a) posterior cruciate ligament (PCL); (b) anterior cruciate ligament (ACL); (c) external lateral ligament (LLE); (d) internal lateral ligament (LLI); (e) meniscus of the tibial plateau to export and repair the surface.

The former procedure is now applied to the development of soft tissues such as the crossed and lateral ligaments that are responsible for maintaining the bone structure and to the meniscus of the tibial plateau (Figure 4).

**Figure 5:**
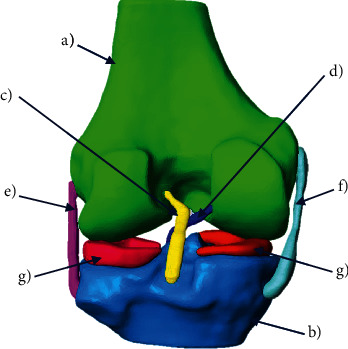
3D biomodel of the knee: (a) cortical bone of the last third of the femur; (b) cortical bone of the tibial plateau; (c) anterior cruciate ligament (ACL); (d) posterior cruciate ligament (PCL); (e) external lateral ligament (LLE); (f) internal lateral ligament (LLI); (g) meniscus.

Finally, the three-dimensional biomodel is produce with all the biological elements necessary for the repair and validation of the model (Figure 5).

All the masks of the elements that produce the three-dimensional biological model from which the .STL files of each structure are generated. It is a model with high biofidelity of the knee, which is composed of cortical bone, trabecular bone, tibial menisci, anterior cruciate ligament, and posterior cruciate ligament. Then, files are exported to a computer program specialized in identifying files of this type, to repair the surface of the model. Later, it goes through the CAD-type computer programs, in which the model is solidified, that is, the surfaces of the model are closed transforming them into a solid entity. This procedure is described below.

For the repair of the surface of the model, CopyCAD® is applied; this is a computer program designed to apply Reverse Engineering, that is to say starting from a scanning of parts, which generate point clouds and convert them into geometries capable of working with any CAD computing tool. This program can solve the details and failures caused by openings in the point cloud and solve them.

**Figure 6:**
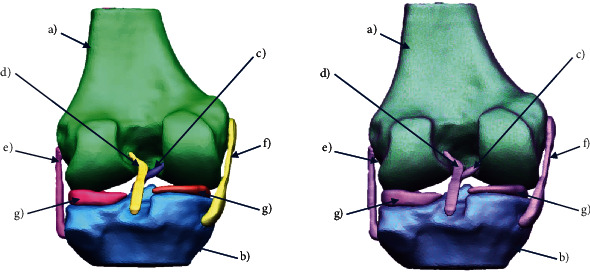
3D biomodel of the knee after repairing the surface: (a) cortical bone of the last third of the femur; (b) cortical bone of the tibial plateau; (c) anterior cruciate ligament (ACL); (d) posterior cruciate ligament (PCL); (e) external lateral ligament (LLE); (f) internal lateral ligament (LLI); (g) meniscus.

A controlled discretization is carried out in each one of the elements of the three-dimensional model. A first controlled discretization is carried out in each one of the elements of the three-dimensional model. This should prevent or hinder a good numerical analysis. This step should be performed on each anatomical structure of the three-dimensional model (Figure 6).

**Figure 7:**
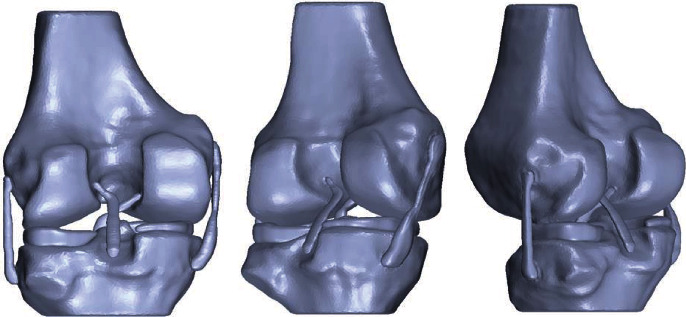
Solidified biomodel of the knee.

Subsequently, once the shell or the corrected surface of all the elements has being obtained, the biomodel is exported to another specialized computer program of the CAD type to solidify the model and store it in Parasolid format (.x_t) (Figure 7).

**Figure 8:**
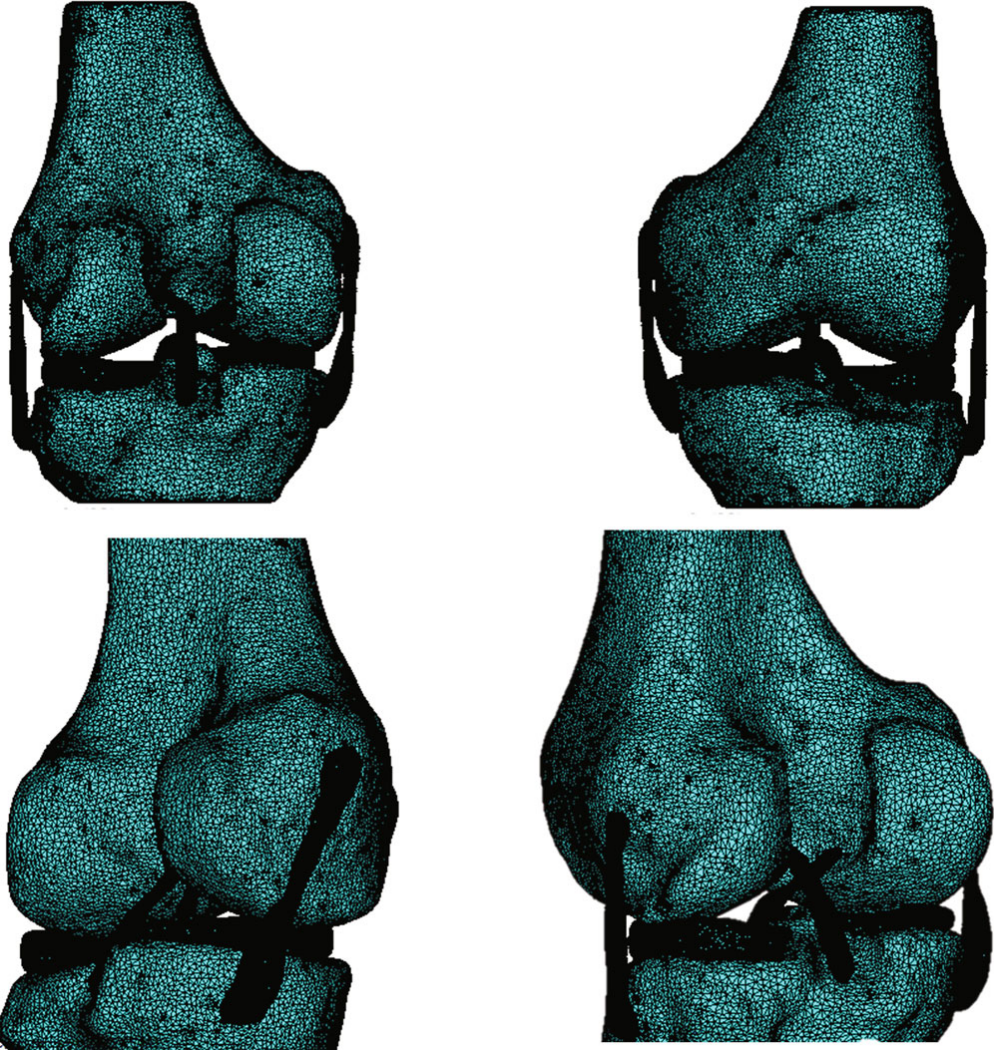
Discretized biomodel in ANSYS®.

Finally, the solid file is exported to a computer tool of FEM to perform the numerical analysis of the three-dimensional model of the knee. As can be seen in Figure 8, the model is discretized in the ANSYS® environment.

## 3. Results

### 3.1. FEM Analysis Considerations

**Figure 9:**
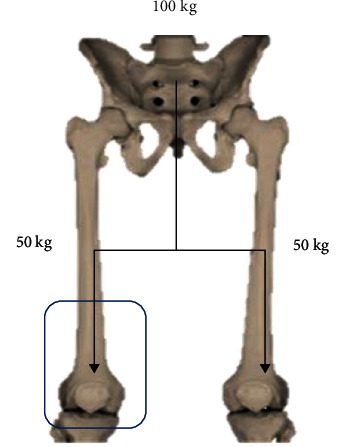
Free body diagram for FEM analysis.

**Figure 10:**
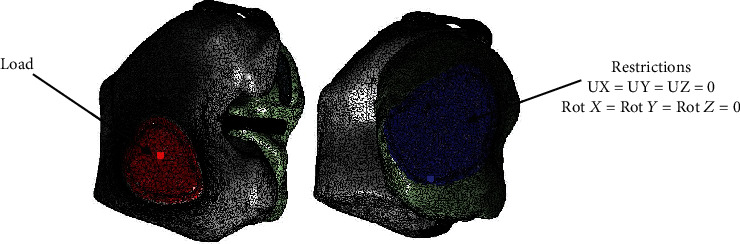
Load application areas and restrictions.

Once the biomodel is obtained and interpreted as a solid by the computer programs, one can proceed to perform the analysis by Finite Element Method. The biomodel has 10 biological tissues, 1,167,404 nodes, and 678,177 elements. A person of 100 kg in a bipodal position was simulated. This weight is distributed in both legs, so the load applied to the model (one knee) is 50 kg [18] (Figure 9). This load will be applied to the cross section of the femur (upper part of the model). The boundary conditions and the restrictions of movement in the sole of the foot, this is transferred to the transverse part of the tibia. This restriction is performed on all axes and their rotations (Figure 10). The analysis meets the characteristics of being elastic, isotropic, continuous, and linear.

Table 1 shows the mechanical properties of the different biological tissues [16] with which it is verified that the Biomodelo can be solved.

In order to simulate the elasto-plastic behavior of each of the tissues, a general model was used, which is based on a kinematic hardening rule with isotropic hardening components comprising the following equations [17, 18]:

(1) $$\begin{array}{c} \text{da}=C\frac{1}{\sigma 0}\left(\sigma -\alpha \right)d{\bar{ɛ}}^{pl}-\gamma \alpha d{\bar{ɛ}}^{pl}, \end{array}$$

(2) $$\begin{array}{c} \sigma 0=\sigma \;|\;0+Q\infty \left(1-e^{-\bar{ɛ}pl}b\right), \end{array}$$

where *ɛ̅pl* is the equivalent plastic unit deformation, *α* is the effort of the lower surfaces, *C* is the kinematic initial module, *γ* is the range in which the kinematic module decreases with respect to the plastic deformation, *σ*0 is the current transfer effort, *σ* | 0 is the original transfer effort, *Q*∞ is the maximum change in yield surface size, and *b* defines the range in which the yield surface changes in relation to the development of the plastic unit deformation.

**Figure 11:**
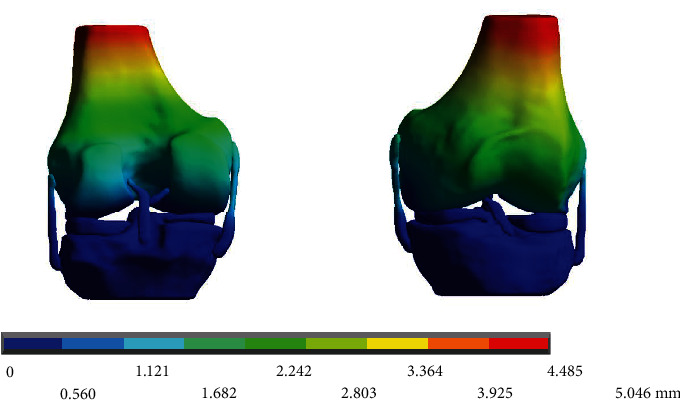
Total deformation of the obtained biomodel.

When performing the numerical analysis, the total deformation of the whole model that originated after receiving the load of the weight of the person is obtained (Figure 11).

This shows that the Biomodel is viable, and FEM programs can solve it. Allowing not only to do the analyses in the biological structure but it is also possible to include in these analyses equipment or mechanical parts such as screws, prostheses, or implants and simulate the behavior of this interaction, allowing to anticipate the scenarios to which they will be subjected and will help to select in a better way than expected.

The biomodels that are obtained have high biofidelity; this means obtaining a more real morphology and morphometry. This attribute will allow; when performing numerical analysis, more real results are obtained. Additionally, this morphological and morphometric precision makes biomodels suitable for prototyping and 3D printing of anatomical structures, with all the advantages that this has. In turn, this facilitates better planning of surgical interventions and even personalizing prostheses or orthotics for each patient [19].

**Figure 12:**
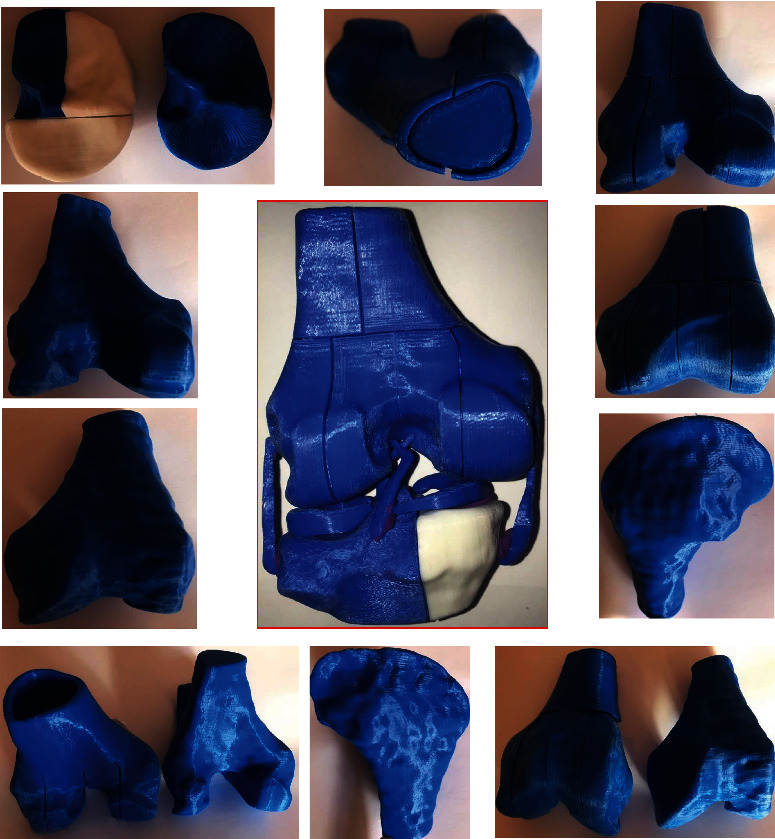
3D printing of knee biomodel (proximal thirds of the femur and tibia) and each of its structures (cortical bone, trabecular bone, meniscus, and ligaments) in real size.

A further advantage of the methodology presented in this work, makes the biomodels generated through it, can be implemented for 3D printing. This impression can be made in the entire biological structure, regardless of size, in each of the tissues that make it up partially or totally, with various matrices in various printers. In this way, print customized tissues, prostheses, and orthotics with a high degree of morphological and morphometric precision (Figure 12).

## 4. Discussion

To obtain an optimal biofidelity in a biomodel, it is necessary to know the whole morphology of the member that will be the effect of study. The knee is one of the most complex structural assemblies of the human body since in it, various elements interact (bones, muscles, ligaments, meniscus). For this reason, it is mandatory to make an accurate model. For this purpose, a computed tomography and/or magnetic resonance is used; in both and the cut section of the limb and densities of each element are fully examined.

The anatomy of the elements that make up the knee such as bones, ligaments, meniscus, and muscles varies for each individual; this is due to the fact that they have been subjected to different external agents throughout their life, as well as the questions of genetics and morphometry of each individual.

It must be considered that the elaboration of a model of this kind requires time and computational resources.

In addition, one must have the necessary knowledge of both the biological part that corresponds to the morphology of each of the anatomical structures and the required computer programs, since the biomodels must be represented in a faithful and most similar way. In this way, it is valid to carefully develop the generation of biomodels with high biofidelity which is an extremely complicated and laborious task. Although it is true, as a wide knowledge of anatomy, biology, and computer systems already mentioned is necessary, to apply the present methodology and facilities this process. In addition, this type of detailed work allows, when performing the analyses with the computational tools of Finite Element Method (FEM), the obtention of results that are more precise for the use in Engineering, Design, and Medicine.

The biomodel carried out in this work with the proposed methodology, in comparison with models made in other works, offers a greater biofidelity. The biomodel obtained considers the various tissues that comprise the organs and faithfully represent its geometry, its morphology, its morphometry, and the characteristic properties of each of the tissues and structures. This allows that, in each one of them, the results obtained, when subjected to analysis, are the reactions most similar to those that would appear in real life. In addition to this, it opens the possibility of generating customized biomodels, where pathological phenomena can be analyzed and the elaboration of treatment and/or surgical and physiotherapy plans.

## 5. Conclusions

Physical injuries represent a very common condition for most people. Any individual, at some time in their life, has experienced an injury. Physical injuries represent a very common condition for most people. Any individual, at some time in their life, has experienced an injury, from a simple sprain to the break of a member. The severity of these injuries varies depending on aspects of the person, age, sex, ethnicity, systemic pathologies, among others, up to the activities that he/she carries out. In most cases, they are usually rehabilitated with a certain degree of simplicity. At other times, the damage can be irreversible, limiting, or even invalidating the individual.

In general, this type of affections is treated and studied by Medicine in its various branches (Traumatology, Orthopedics, Physiotherapy, Sports Medicine, Physical Rehabilitation and Prostheses mainly). However, the relationship that this area of science has established with engineering has allowed the finding of new means of rehabilitation, equipment, materials and therapies. This linkage allowed the emergence of new branches of these sciences such as Bioengineering, Biotechnology, Biomedical Engineering, Biomechanics, and in recent times, Tissue Engineering, and Mechanobiology.

This has been made it possible thanks to the interaction between the biological areas and the tools used by Engineering and Mathematics. One of these tools is the analysis by means of the Finite Element Method.

Among the most notable interactions is the use of a study that is used for the diagnosis of physical injuries, the computed tomography and magnetic resonance. The quality of the images acquired through this medium and the development of new CAD/CAM computer programs allows the generation of three-dimensional computational models of the different anatomical structures called Biomodels. These models allow a better visualization of the structures of interest and the possibility of being able to carry out on them; the numerical analyses before the various phenomena act on the structures and prevent considerable damage consequences.

There are many works where various analyses are carried out through the FEM, in different organs and tissues of the human body. Most of these analyses are performed on hard tissues such as the jaw [20], craneal bones [21], vertebrae [22], and even smaller anatomical structures but with great geometric complexity such as the teeth [18]. Soft tissue analyses are performed in smaller numbers, but it is possible to find analyses of the ligaments [23], meniscus [24], veins [25], brain, heart [26], among others. Unfortunately, papers that present a conjunction of the analysis in both tissues are few. There are some made in very small anatomical structures such as the teeth where three different tissues (two hard and one soft) [18] or the knee, which is a large structure.

In these analyses, it is possible to observe the bones as hard tissues, as well as ligaments and menisci as soft tissues [27] [28]. However, in these works, the bone is considered as solid and does not present differentiation between the two types of osseous tissue that are in real life. With the passage of time, these works have improved their morphological conditions with which it is intended to obtain more real results. However, with the methodologies that were used, they could not have the real morphology of the tissues.

## Figures and Tables

**Table 1:** Material properties.

Tissue	Young's module (MPa)	Poisson's ratio
Cortical bone	15 000	0.32
Trabecular bone	100	0.3
ACL	64	0.45
PCL	67	0.45
Lateral ligament	61	0.45
Meniscus	55	0.3

## Data Availability

All data generated or analyzed during this study are included in this published article.
